# Clear Cell Acanthoma with Melanophages Simulating a Spitz Nevus

**DOI:** 10.5826/dpc.1103a89

**Published:** 2021-07-08

**Authors:** Thaís Andrade de Oliveira, Gabriella Brancaccio, Ilenia D’Ambra, Andrea Ronchi, Francesca Pagliuca, Giuseppe Argenziano

**Affiliations:** 1 Department of Dermatology, Faculdade de Medicina de Jundiaí, São Paulo, Brazil; 2 Dermatology Unit, University of Campania “L. Vanvitelli”, Naples, Italy; 3 Pathology Unit, Department of Mental and Physical Health and Preventive Medicine, University of Campania “L. Vanvitelli”, Naples, Italy

**Keywords:** Dermoscopy, skin neoplasm, pigmented clear cell acanthoma, pathology

## Introduction

Clear cell acanthoma (CCA) is an unusual and benign epidermal tumor that is usually found on the limbs as a solitary reddish papule. Other variants include polypoid, giant, multiple, eruptive, and pigmented clear cell acanthoma. The typical dermoscopic pattern, known as “string of pearls”, is characterized by dotted and glomerular vessels in a mesh-like or serpiginous arrangement [1]. Here, we describe a clear cell acanthoma with melanophages, dermoscopically mimicking a Spitzoid melanocytic tumor.

## Case Presentation

**Figure 1:**
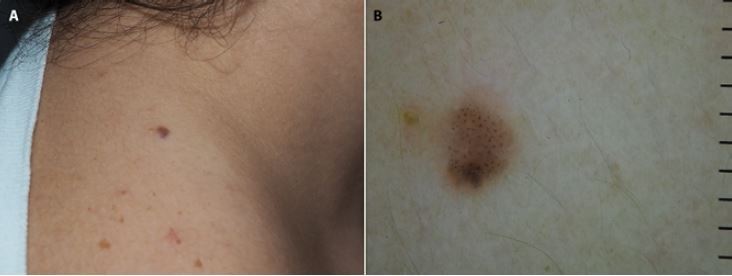
(A) A slightly elevated, brownish, oval papule measuring about 3 × 4 mm located on the right shoulder. (B) On dermoscopy, the lesion showed brown globules with regular distribution, and a kind of a blotch at 6 hours.

**Figure 2:**
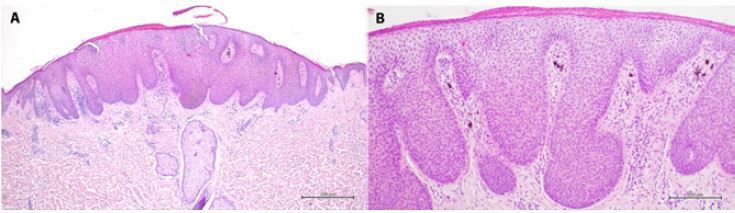
(A) Epithelial tumor showing psoriasiform hyperplasia, parakeratosis, and pale cytoplasm of keratinocytes (H&E, ×40). (B) Some melanophages were present in the superficial dermis (H&E, ×100).

A 22-year-old woman presented a slightly elevated, brownish, oval papule measuring 3 × 4 mm located on her right shoulder (Figure 1A). The dermoscopic findings were equivocal, revealing brown globules with regular distribution and a brown blotch at the periphery (Figure 1B). The first hypothesis was that of a Spitz Nevus, and excision was indicated. Histological examination (H&E stain) revealed an epithelial tumor characterized by psoriasiform hyperplasia, parakeratosis, and large keratinocytes with pale cytoplasm. Some melanophages were evident at the level of the superficial dermis (Figure 2). Thus, a clear cell acanthoma (CCA) with melanophages was diagnosed.

## Discussion

Pigmented CCA was first described by Langer et al [2], who reported 5 cases of macroscopically pigmented forms. Microscopically, the authors found pale glycogen-containing keratinocytes, increased number of melanocytes containing melanin granules among the tumoral keratinocytes and, most importantly, large melanophages clumps between tumor nests in papillary dermis, characteristic of the pigmented variant. In the present case, the lesion appeared clinically pigmented due to the presence of melanophages in papillary dermis, rather than the presence of melanocytes in the context of the lesion.

Overall, 12 cases of pigmented CCA have been described in the literature. Scheinfeld [1], reported a glistening brown nodule which was diagnosed as a pigmented CCA, and Jacyk et al [2], reported a case of a man with multiple pigmented CCA, 4 in total. Both reports lacked a dermoscopic appearance description. Later, Saeki et al [1], described a pigmented CCA located on the finger. This was dermoscopically characterized by a homogeneous brown-to-black pigmentation with peripheral regular network, simulating a pigmented nevus. Finally, Kiyohara et al [1], published the first case of a pigmented CCA with a string of pearls pattern visible on dermoscopy. Table 1 shows the dermoscopic descriptors that have already been reported in the literature, including ours.

In conclusion, pigmented CCA is a rare variant of an uncommon benign tumor with unspecific dermoscopic features, whose diagnosis is usually done relying on histological findings.

## Figures and Tables

**Table 1:** Pigmented CCA: dermoscopic descriptors

AUTHORS	DERMOSCOPY DESCRIPTORS
Saeki et al, 2014	Homogeneous brown-to-black pigmentation with peripheral regular network.
Kiyohara et al, 2019	Homogeneous brown pigmentation with a peripheral dark red area, that corresponds to a string of pearls pattern.
Oliveira et al, 2020	Brown globules with regular distribution over homogeneous brown pigmentation.
